# Effects of Liraglutide Combined with Short-Term Continuous Subcutaneous Insulin Infusion on Glycemic Control and Beta Cell Function in Patients with Newly Diagnosed Type 2 Diabetes Mellitus: A Pilot Study

**DOI:** 10.1155/2016/6839735

**Published:** 2015-11-10

**Authors:** Weijian Ke, Liehua Liu, Juan Liu, Ailing Chen, Wanping Deng, Pengyuan Zhang, Xiaopei Cao, Zhihong Liao, Haipeng Xiao, Jianbin Liu, Yanbing Li

**Affiliations:** Department of Endocrinology and Diabetes Center, The First Affiliated Hospital of Sun Yat-sen University, Guangzhou 510080, China

## Abstract

The objective of this paper is to investigate the effects of liraglutide in combination with short-term continuous subcutaneous insulin infusion (CSII) therapy on glycemic control and beta cell function in patients with newly diagnosed type 2 diabetes mellitus (T2DM). Thirty-nine eligible newly diagnosed T2DM patients were recruited and randomized to receive either of two therapies: short-term CSII alone (CSII alone group) or CSII in combination with liraglutide (CSII + Lira group) for 12 weeks. Blood glucose control, homeostasis model assessment (HOMA) indices, and acute insulin response (AIR) were compared between the two groups. The patients in CSII + Lira group achieved euglycemia with equivalent insulin dosage in shorter time (1 (0) versus 2 (3) days, *P* = 0.039). HbA1c at the end of study was comparable between two groups (6.3 ± 0.7% versus 6.0 ± 0.5%, for CSII alone group and CSII + Lira group, resp., *P* = 0.325). The increment of AIR was higher in CSII + Lira group (177.58 (351.57) *μ*U·min/mL versus 58.15 (51.30) *μ*U·min/mL, *P* < 0.001). However, after stopping liraglutide, its effect on beta cell function disappeared completely. Liraglutide combined with short-term CSII was effective in further improving beta cell function, but the beneficial effects did not sustain after suspension of the therapy.

## 1. Introduction

Diabetes mellitus is the most common metabolic disease and becomes a heavy burden of public health systems. In China, the prevalence of diabetes and prediabetes in adults was 11.6% and 50.1%, respectively [1]. Deterioration of beta cell function and insulin resistance are two fundamental pathophysiologic defects of type 2 diabetes mellitus (T2DM). It has been proven that at the time when T2DM was established, the loss of beta cell function was shown to reduce by 50% and this decline of beta cell function progressed over time although traditional antihyperglycemic therapy had been applied [2]. In order to postpone the progress of disease, new therapies are required to persistently act on beta cell failure and insulin resistance.

In our previous studies, intensive insulin interventions, especially continuous subcutaneous insulin infusion (CSII), induced near-normoglycemia over 1 year without antihyperglycemic agents in nearly half of the patients with newly diagnosed T2DM with favorable recovery of beta cell function [3, 4]. The reason for glycemic remission in these patients was considered to be alleviation of glucotoxicity, lipotoxicity, and insulin resistance [5, 6]. However, the therapy, which lasted for only 2-3 weeks, had its limitations in covering the multiple pathophysiological defects in the long term. In another trial investigating the effect of combination of metformin or rosiglitazone with CSII, the combination of metformin for 3 months had better effects on insulin secretion function measured by acute insulin response (AIR) and HOMA-B while the combination with rosiglitazone better improved muscle insulin resistance [7]. Since the two medicines used in that study mainly were targeted at insulin resistance, it would be of great interest whether combining CSII with medicine intervening beta cell failure, the critical pathophysiology mechanism of T2DM, might provide better clinical outcomes compared with short-term CSII alone.

Liraglutide, a glucagon-like peptide-1 (GLP-1) analog with a 97% homology with endogenous GLP-1, lowers blood glucose by enhancing glucose-dependent insulin secretion of beta cells and suppressing glucagon secretion of alpha cells [8]. In some rodent studies, liraglutide reduced beta cell apoptosis and promoted its proliferation, which might potentially modify the progression of T2DM [9, 10]. Moreover, liraglutide also reduced body weight in a dose dependent manner, ameliorated lipid profiles, lowered blood pressure [11], and reduced cardiovascular risk markers such as adipokines and proinflammatory factors [12], all of which are favorable in management of T2DM. We hypothesized that combining CSII with liraglutide might have better effects over CSII alone. Therefore, we conducted this randomized controlled trial investigating whether liraglutide in combination with short-term CSII therapy has better effect over CSII alone on beta cell function and sustained glycemic control.

## 2. Subjects and Methods

### 2.1. Subjects

Thirty-nine newly diagnosed T2DM patients diagnosed according to the 1999 World Health Organization diagnostic criteria [13], without previous usage of antihyperglycemic and antihyperlipidemic medication, were enrolled. The included patients were between 20 and 65 years of age and had a body mass index of 20–35 kg/m^2^, with fasting plasma glucose (FPG) between 7.0 and 16.7 mmol/L. Patients were excluded if they had severe acute or severe chronic diabetic complications and severe intercurrent illness and were positive for autoimmune antibodies against islets or with a recent history of being treated with corticosteroid, immunosuppressing drugs, or cytotoxic drugs.

### 2.2. Study Design

All patients were admitted to the hospitals after a 3–5-day run-in period and assigned to one of the following two groups by sequentially opening sealed, opaque envelopes arranged in a computer-generated random order. During hospitalization, patients in CSII alone group received insulin aspart (NovoRapid, Novo Nordisk, Bagsværd, Denmark) or insulin lispro (Humalog, Eli Lilly, USA) with an insulin pump (MiniMed 712, Medtronic, Northridge, CA) as CSII therapy, while the CSII + Lira group received liraglutide (Victoza, Novo Nordisk, Bagsvaerd, Denmark) 0.6 mg per day in addition to aforementioned CSII regimen. The initial insulin dosage was 0.5–0.7 IU/kg/d, with the total daily dosage divided into 50/50 as basal and bolus infusion. In order to achieve euglycemia, basal rates and premeal boluses of insulin were adjusted every day according to capillary blood glucose values which were monitored at least 7 times per day. The glycemic goal was defined as fasting blood glucose less than 6.0 mmol/L and postprandial blood glucose less than 8.0 mmol/L. After the glycemic targets were achieved, CSII treatments were maintained for additional 14 days. After being discharged from the hospital, all patients were guided with diet and physical exercise. Patients in CSII + Lira group continued to use liraglutide 1.2 mg per day until the 12-week treatment period was finished.

All recruited patients provided written informed consent for participation, and the study protocol was approved by the Medical Research and Ethics Committee of the First Affiliated Hospital of Sun Yat-sen University (Guangzhou, China). This study is registered at ClinicalTrials.gov with trial registration identifier number of NCT01471808.

### 2.3. Measurements

Baseline anthropometric data such as blood pressure, height, weight, and waist and hip circumferences were measured, while fasting blood samples were collected for measurements of FPG and HbA1c. An intravenous glucose tolerance test (IVGTT) using 25 g of glucose (50 mL of 50% glucose) was conducted to assess AIR which was used to estimate the first-phase beta cell insulin secretion. Serum insulin levels before and 1, 2, 4, 6, and 10 min after glucose injection were measured, and AIR was calculated as the incremental trapezoidal area during the first 10 min of the IVGTT. PPG (after breakfast) levels were evaluated on the previous day. Homeostasis model assessment was used to estimate insulin resistance (HOMA-IR) and beta cell function (HOMA-B). HOMA-IR = FPG × fasting insulin/22.5. HOMA-B = 20 × fasting insulin/(FPG − 3.5). Daily insulin dosage of each patient was recorded. After CSII suspension, all baseline measurements were repeated at least 15 hours after cessation of insulin infusion and before liraglutide injection for CSII + Lira group. At the 12-week visit, the assessments were performed after 12 weeks of CSII suspension for CSII alone group or 7 days after liraglutide suspension.

## 3. Statistical Analyses

Data were analyzed with SPSS software for Windows version 16.0. Normally distributed data were presented as mean ± SD, and nonnormally distributed variables (triglyceride, AIR, HOMA-B, and HOMA-IR) were expressed as median (interquartile range). The differences of normally distributed data between two groups were compared by independent-sample *t*-tests, while the comparisons of nonnormally distributed variables were using Mann-Whitney *U* tests. Paired-sample *t*-tests or Wilcoxon signed ranks tests were performed to estimate the changes before and after intervention. The *χ* ^2^ tests were applied to analyze the differences of proportions. A 2-sided value of *P* < 0.05 was defined statistically significant.

## 4. Results

### 4.1. Baseline Characteristics

The enrolled patients were 45.91 ± 8.7 years in age, with a BMI of 25.7 ± 2.8 kg/m^2^, FPG of 11.4 ± 3.2 mmol/L, PPG of 17.4 ± 5.9 mmol/L, and HbA1c of 10.7 ± 2.2%. They were assigned to CSII alone group (*n* = 19) and CSII + Lira group (*n* = 20) and finished CSII therapy. At the subsequent 12-week visit 8 patients (20.5%, 4 in CSII alone group, 4 in CSII + Lira group) dropped out due to withdrawal of consent. At baseline there were no significant differences in clinical characteristics, FPG, and HbA1c between two groups except for PPG, which was slightly higher in CSII + Lira group (15.2 ± 6.1 mmol/L versus 14.4 ± 4.1 mmol/L, *P* = 0.025). Markers of beta cell function (AIR and HOMA-B) and insulin sensitivity (HOMA-IR) were also comparable (Table 1).

### 4.2. CSII Therapy

**Figure 1:**
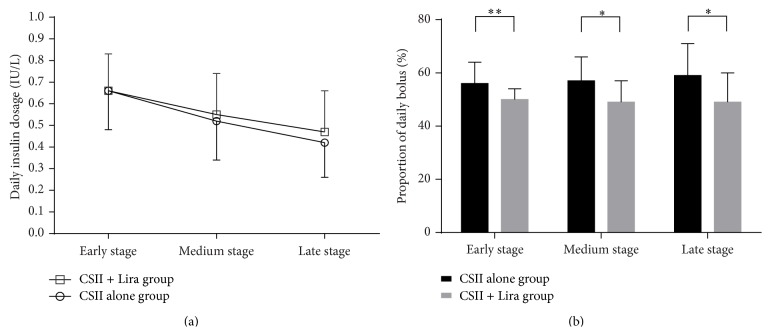
Daily insulin dosage and proportion of daily bolus of two groups. ((a) Daily insulin dosage, (b) proportion of daily bolus, ^*∗*^ *P* < 0.05, ^*∗∗*^ *P* < 0.01.)

All patients achieved euglycemia in the first week of CSII treatments. Patients in CSII + Lira group reached target glycemic control in less time than those in CSII alone group (2 (3) days versus 1 (0) days, for CSII alone group and CSII + Lira group, resp., *P* = 0.039). After achieving euglycemia, daily insulin dosages decreased gradually. The 14 days of CSII for maintaining euglycemia was divided into three stages: early stage (days 1–5), medium stage (days 6–10), and late stage (days 11–14). Average daily insulin dosage was similar in both groups, while the proportions of daily bolus dosage in total daily insulin dosage were lower in CSII + Lira group throughout the CSII therapy (Figure 1).

### 4.3. Beta Cell Function

**Figure 2:**
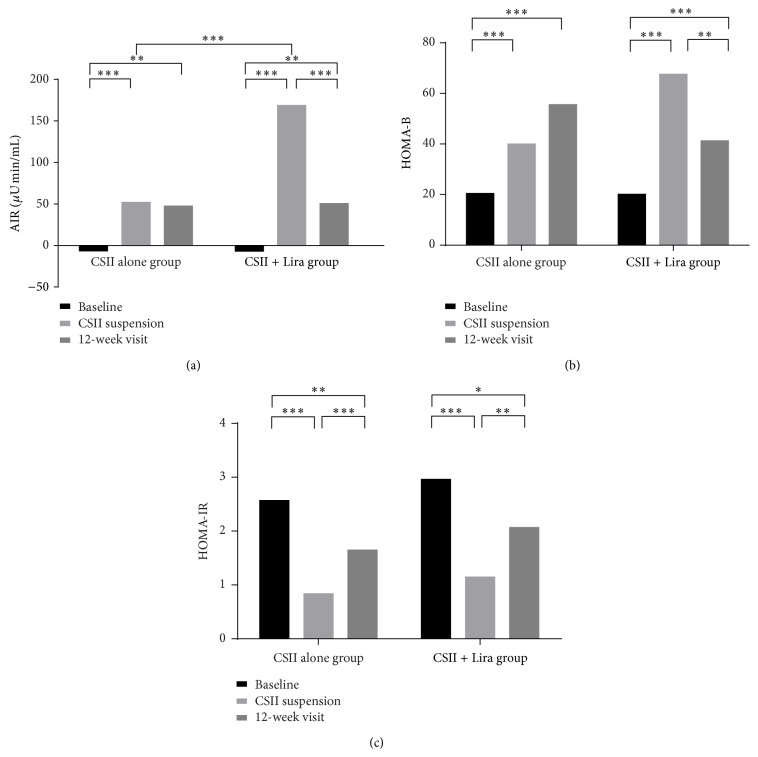
Beta cell function and insulin sensitivity of two groups, (a) AIR of baseline and after intervention, (b) HOMA-B of baseline and after intervention, and (c) HOMA-IR of baseline and after intervention, ^*∗*^ *P* < 0.05, ^*∗∗*^ *P* < 0.01, and ^*∗∗∗*^ *P* < 0.001.

AIR was restored in all patients after short-term CSII therapy compared with baseline. At CSII suspension, AIR improved from −6.60 (26.2) *μ*U·min/mL to 52.05 (100.55) *μ*U·min/mL in CSII alone group and from −6.98 (21.71) *μ*U·min/mL to 168.62 (350.95) *μ*U·min/mL in CSII + Lira group. The increment of AIR was significantly higher in CSII + Lira group than that in CSII alone group (177.58 (351.57) *μ*U·min/mL versus 58.15 (51.30) *μ*U·min/mL, *P* < 0.001). However, after withdrawal of liraglutide after the 12-week treatment, the improvement in AIR rapidly disappeared in CSII + Lira group (168.62 (350.95) *μ*U·min/mL versus 50.43 (70.40) *μ*U·min/mL, for CSII suspension and 12-week visit, resp., *P* < 0.001). Therefore, AIR between two groups at the end of follow-up was similar (*P* = 0.921) (Figure 2(a)). In both groups, HOMA-B was ameliorated significantly after CSII treatment compared with baseline. Similar to AIR, HOMA-B in CSII + Lira group was higher than that in CSII alone group at the end of CSII (67.64 (46.31) versus 40.00 (35.53), *P* = 0.007), but the improvement was not sustained after stop of liraglutide at 12-week visit (41.28 (21.62), *P* = 0.003, compared with that after CSII suspension) and became similar to CSII alone group (55.65 (56.27), *P* = 0.110) (Figure 2(b)).

### 4.4. Insulin Resistance

HOMA-IR decreased significantly after CSII compared with baseline in both groups. But at 12-week visit, HOMA-IR was significantly elevated from CSII suspension in both groups (Figure 2(c)).

### 4.5. Glycemic Control

**Figure 3:**
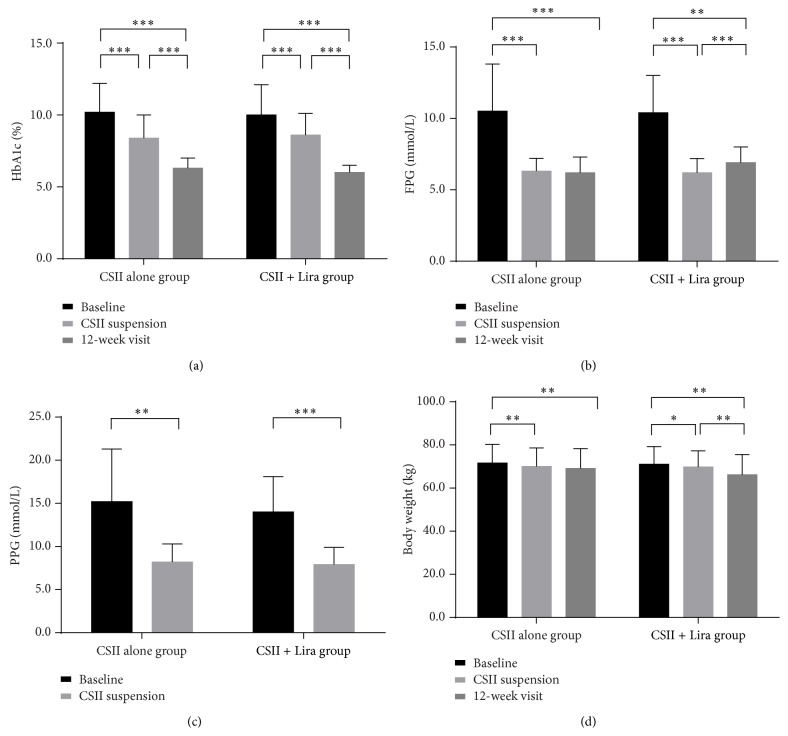
Glycemic control and body weight of two groups. (a) HbA1c of baseline and after interventions, (b) FPG of baseline and after interventions, (c) PPG of baseline and after CSII suspension, and (d) body weight of baseline and after intervention, ^*∗*^ *P* < 0.05, ^*∗∗*^ *P* < 0.01, and ^*∗∗∗*^ *P* < 0.001.

HbA1c level was slightly lower in CSII + Lira group at the end of the 12-week follow-up compared with CSII alone group but did not reach statistical significance (6.0 ± 0.5% versus 6.3 ± 0.7%, *P* = 0.325), with similar proportions of patients who achieved HbA1c ≤ 6.5% (73% (11/15) versus 94% (15/16), for CSII alone group and CSII + Lira group, resp., *P* = 0.146) (Figure 3(a)). Considerable reduction in FPG and PPG from baseline was observed at CSII suspension. However, at 12-week visit there was a slight but statistically significant elevation of FPG in CSII + Lira group from CSII suspension (from 6.1 ± 0.9 mmol/L to 6.9 ± 1.1 mmol/L, *P* = 0.01), which was not seen in CSII alone group (Figure 3(a)). There was a tendency of higher hyperglycemia relapse rate (>7.0 mmol/L) in CSII + Lira group at 12-week visit (20% (3/15) versus 43.75% (7/16), for CSII alone group and CSII + Lira group, resp., *P* = 0.208) (Figure 3(b)).

### 4.6. Body Weight

At CSII suspension, certain body weight loss was recorded in both groups (−1.6 ± 2.0 kg versus −1.2 ± 2.3 kg, for CSII alone group and CSII + Lira group, resp., *P* = 0.574). Continuous decline of body weight during the 12-week visit was recorded in CSII + Lira group (69.8 ± 7.5 kg versus 66.2 ± 9.3 kg, for CSII suspension and 12-week visit, resp., *P* = 0.005) but not in CSII alone group; however, the reduction of body weight during the 12-week visit in the two groups did not reach statistical significance (−1.6 ± 3.5 kg versus −3.3 ± 4.1 kg, for CSII alone group and CSII + Lira group, resp., *P* = 0.207) (Figure 3(d)).

## 5. Safety Issues

During short-term CSII therapy phase, the incidence of hypoglycemia which was defined as capillary blood glucose level <3.9 mmol/L was similar in CSII alone group and CSII + Lira group (4 (5) versus 2 (3) times per patient, *P* = 0.120). Most of the hypoglycemic episodes were mild and could be corrected after ingestion of carbohydrate. No severe hypoglycemia was recorded in either group. Gastrointestinal symptoms happened in 35% of patients in CSII + Lira group in the first few days of liraglutide injections, and most of these symptoms were well tolerated. No hypoglycemic events were reported after CSII suspension.

## 6. Discussion

Intensive insulin treatment was introduced in the management of newly diagnosed T2DM since 1997 [14]. By fast correction of glucotoxicity and lipotoxicity, intensive insulin treatment is able to induce long-term glycemic remission and thereby be suggested by the latest Chinese guideline for T2DM [3, 4, 15]. In this study, a GLP-1 analog, liraglutide, was used as an add-on therapy of CSII and lasted for additional 12 weeks. As expected, liraglutide facilitated the achievement of euglycemia by shortening the time required for insulin dose titration before reaching glycemic targets. Liraglutide was also reported to reduce the daily insulin requirement in patients with more advanced T2DM treated with insulin [16]. Although the total daily insulin dosage throughout CSII treatment did not significantly differ between the two treatment groups, liraglutide significantly decreased the proportion of daily premeal bolus. Furthermore, there was also a tendency of better average glycemic control in CSII + Lira group during the 12-week extended therapy phase, as indicated by a lower HbA1c level than that in CSII alone group at the end of the follow-up. These findings were probably attributed to a better amelioration of beta cell function in CSII + Lira group compared with that in CSII alone group. These data were in accordance with previous reports on liraglutide, which showed that it reduced hyperglycemia, especially postprandial glycemic fluctuation, by glucose-dependent insulinotropic effect [17].

However, to our surprise, shortly after the suspension of liraglutide, its effect on beta cell function rapidly faded with 1 week, leading to an elevation of fasting blood glucose. The underlying mechanism for the worsening of clinical parameters remains unknown. Recently Retnakaran et al. reported that 48 weeks of liraglutide administration in patients with mean diabetes duration of 2-3 years after 4 weeks of insulin therapy also robustly increased beta cell function measured by ISSI-2 [18]. Similar to this study, they also found a rapid deterioration of beta cell function shortly after cessation of liraglutide. However, an earlier observation showed that, in patients whose blood glucose was insufficiently controlled by metformin, a prolonged treatment with exenatide for 3 years had a slight but statistical significant benefit in beta cell function 4 weeks after stopping the medicine, which was not seen in the 1-year follow-up. There are several possible explanations for the discrepancy between short-term and long-term GLP-1 analogs therapies. Firstly, because of the beneficial effects of GLP-1 analogs on beta cell proliferation and apoptosis from rodent models, liraglutide was expected to further improve functional beta cell mass [9, 10]. However, the renewal rate of beta cells in human islets was so slow that a prolonged therapy targeted at pancreatic beta cells might be necessary for an overt change in islet architecture. Less than 1 year, according to the results from both Retnakaran et al. [18] and this study, was not enough. Secondly, part of the effects of GLP-1 analogs is attributed to their effect on body weight which could help to relieve insulin resistance and restore beta cell function. In LEAD-3 monostudy, the maximum weight loss in 1.8 mg liraglutide treatment group existed in 20 weeks [11]. Although ongoing weight loss was observed in CSII + Lira group rather than CSII alone group in this study, the difference of weight loss between the two groups was not statistically significant in a relatively short treatment period (12 weeks) in a lower dose (1.2 mg/d). Thirdly, it has been well documented that GLP-1 analogs could suppress inappropriate secretion of glucagon from alpha cells. However, despite certain controversy, there are some reports showing that incretin therapy may induce hyperplasia of pancreatic alpha cells in human and rodent models [19–21]. The importance and clinical consequence of alpha cell hyperplasia are largely unknown due to lack of data, but it is not impossible that, after stopping liraglutide, the previous suppressed glucagon secretion could rebound, resulting in relapse of hyperglycemia.

Previous observations suggested that persistent improvement in insulin sensitivity was critical for long-term maintenance of near-normoglycemia [5, 6]. Our previous studies also showed that combining CSII with insulin sensitizers, that is, metformin or rosiglitazone, increases short-term remission rate by improving both insulin sensitivity and beta cell function patients with newly diagnosed T2DM. As shown in this study, 12-week treatment with liraglutide was not sufficient to cause prominent effect on insulin resistance. Enhancement of insulin action may decrease insulin demand and subsequent beta cell overload, endoplasmic reticulum stress, or oxidative stress, leading to a longer duration of glycemic remission [22, 23]. In this point of view, insulin sensitizer, other than insulin secretagogues, should be tested as combination therapy to CSII in future studies.

There were several limitations in this study. First of all, as a pilot study, the relatively small sample size may reduce the statistical power when analyzing some clinical parameters. Second, IVGTT and homeostasis model were used to evaluate beta cell function and insulin resistance. Using clamp technique as well as physiologic challenge tests such as OGTT or mix-meal test may provide further useful information.

In conclusion, liraglutide in combination with CSII could facilitate the achievement of glycemic targets and further improve beta cell function in patients with newly diagnosed T2DM. Rapid waning of beneficial effects of liraglutide implied that a prolonged treatment period might be required to obtain a sustained favorable outcome.

## Figures and Tables

**Table 1:** Baseline characteristics of patients.

Characteristic	Group 1	Group 2	*P* value
Number	19	20	0.127
Gender (F/M)	4/15	5/15	0.770
Age (years)	42.1 ± 7.6	42.3 ± 9.9	0.127
Family history (with/without)	10/9	10/10	0.869
Blood pressure (mmHg)			
Systolic	116.5 ± 12.3	118.2 ± 11.7	0.914
Diastolic	74.4 ± 9.9	77.4 ± 12.0	0.372
Weight (kg)	71.6 ± 8.7	71.0 ± 8.2	0.441
BMI (kg/m^2^)	25.5 ± 2.4	25.4 ± 2.8	0.743
Waist circumference (cm)	89.0 ± 6.9	89.4 ± 7.9	0.869
Waist to hip ratio	1.07 ± 0.62	0.94 ± 0.07	0.088
HbA1c (%)	10.2 ± 2.0	10.0 ± 2.1	0.862
FPG (mmol/L)	10.5 ± 3.3	10.4 ± 2.6	0.130
PPG (mmol/L)	15.2 ± 6.1	14.4 ± 4.1	0.025
Triglyceride (mmol/L)	1.80 (1.50)	1.43 (1.69)	0.899
CHOL (mmol/L)	5.1 ± 0.9	5.2 ± 1.1	0.408
LDL-c (mmol/L)	3.33 ± 0.83	3.52 ± 0.71	0.461
HDL-c (mmol/L)	1.18 ± 0.46	1.09 ± 0.19	0.099
AIR (*μ*U·min/mL)	−6.60 (26.2)	−6.98 (21.71)	0.911
HOMA-IR	2.57 (2.78)	3.96 (2.71)	0.258
HOMA-B	20.48 (16.46)	20.16 (26.15)	0.584
